# Regulation of Plant Genes with Exogenous RNAs

**DOI:** 10.3390/ijms26146773

**Published:** 2025-07-15

**Authors:** Alexandra S. Dubrovina, Andrey R. Suprun, Konstantin V. Kiselev

**Affiliations:** Laboratory of Biotechnology, Federal Scientific Center of the East Asia Terrestrial Biodiversity, Far Eastern Branch of the Russian Academy of Sciences, Vladivostok 690022, Russia; suprun@biosoil.ru (A.R.S.); kiselev@biosoil.ru (K.V.K.)

**Keywords:** exogenous RNAs, dsRNA, siRNA, miRNA, external application, plant gene regulation, gene silencing, RNA interference

## Abstract

Exogenous RNA application, also known as spray-induced gene silencing (SIGS), is a new approach in plant biotechnology that utilizes RNA interference (RNAi) to modify plant traits. This technique involves applying RNA solutions of double-stranded RNA (dsRNA), hairpin RNA (hpRNA), small interfering RNA (siRNA), or microRNA (miRNA) directly onto plant surfaces. This triggers RNAi-mediated silencing of specific genes within the plant or invading pathogens. While extensively studied for enhancing resistance to pathogens, the application of exogenous RNA to regulate plant endogenous genes remains less explored, creating a rich area for further research. This review summarizes and analyzes the studies reporting on the exogenously induced silencing of plant endogenes and transgenes using various RNA types. We also discuss the RNA production and delivery approaches, analyze the uptake and transport of exogenous RNAs, and the mechanism of action. The analysis revealed that SIGS/exoRNAi affects the expression of plant genes, which may contribute to crop improvement and plant gene functional studies.

## 1. Introduction

External RNA application for RNA interference (RNAi) induction is a novel, transgene-free approach in plant biotechnology and agriculture [1,2,3]. It offers a solution for transgene-free plant protection and trait manipulation. This technique, known as spray-induced gene silencing (SIGS) or exogenous RNA interference (exoRNAi), shows promise for plant protection, yield improvement, and other modifications [4,5,6]. Unlike traditional genetic engineering methods, which permanently alter the plant genome, SIGS utilizes the natural RNAi pathway—a crucial defense mechanism against viral infections and transposable element movement [7,8]. RNAi regulates different processes in plants, such as plant development, growth regulation, reproduction, and stress tolerance [9,10,11,12]. In SIGS, plant surfaces are treated with solutions containing key RNAi molecules, such as double-stranded RNAs (dsRNAs), small interfering RNAs (siRNAs), hairpin RNAs (hpRNAs), and microRNAs (miRNAs). These RNA molecules are designed to target specific genes in the plant or invading pathogens. Plant cellular machinery recognizes these RNAs and uses them as blueprints to guide the RNAi machinery in degrading specific mRNA molecules [13,14].

RNAi is a complex and multifaceted process central to gene regulation in various organisms, including plants, that functions to downregulate or silence genes involved in different processes [12,15,16]. The mechanism of RNAi, broadly defined, involves the silencing of gene expression via mRNA degradation or translational repression [14,17,18,19]. RNAi is mediated by dsRNAs as precursors or hairpin-like RNA structures processed from the folding of self-complementary RNAs [14,17,18,19]. RNAi, often referred to as post-transcriptional gene silencing (PTGS), is also implicated in triggering transcriptional gene silencing (TGS) [13,20,21]. TGS involves the recruitment of chromatin-modifying enzymes to the promoter region of the target genes, leading to epigenetic modifications that render the chromatin structure less accessible to the transcriptional machinery. This effectively silences gene expression at the transcriptional level.

The RNAi-inducing dsRNAs or hpRNA-like structures originate from various sources, including exogenous sources (e.g., viruses and their replication intermediates) or endogenous transcripts (e.g., miRNA precursors or others), which act as initiators of the intricate RNAi pathway. The crucial distinction between siRNAs and miRNAs lies primarily in their biogenesis [18,22]. siRNAs are generally derived from longer dsRNAs, often originating from viral infections or experimental introduction, whereas miRNAs are transcribed from endogenous genes as larger primary transcripts (pri-miRNAs) by RNA polymerase II. These pri-miRNAs undergo processing, generating precursor miRNAs (pre-miRNAs), which are then exported to the cytoplasm for further processing [22,23]. The cleavage of the dsRNAs or pre-miRNAs is performed by RNase III enzymes, often referred to as Dicer-like (DCL) proteins [24]. These enzymes cleave the precursors into shorter, more manageable fragments known as siRNAs or miRNAs, depending on their origin and processing pathway. Once generated, both siRNAs and miRNAs are incorporated into the RNA-induced silencing complex (RISC) where they guide the complex to target mRNAs. The RISC then triggers either mRNA degradation or translational repression of the target mRNAs [22]. The size and structure of these small RNAs influence their target specificity and the type of silencing they induce [25]. For instance, miRNAs often regulate gene expression through translational repression, while siRNAs predominantly trigger mRNA degradation.

Recent research has explored the potential of using the phenomenon of RNAi for crop improvement and plant protection through several approaches, such as host-induced gene silencing (HIGS), virus-induced gene silencing (VIGS), or SIGS. VIGS utilizes modified plant viruses as delivery vectors for dsRNA [26]. HIGS takes advantage of the RNA trafficking from plant hosts to interacting pathogens. It involves engineering plants to produce pathogen-specific dsRNAs designed to silence vital genes in the invading pathogen [27,28]. These dsRNAs are then transported to the invading pathogen and lead to the silencing of a vital pathogen target. Plants can also be genetically engineered to produce dsRNA that targets different endogenous genes, thereby improving other crop properties such as tolerance to abiotic stress or growth [29]. SIGS involves the exogenous addition of pathogen-specific siRNAs or dsRNAs to plants, which can enhance resistance to viral, fungal, and insect attacks. HIGS, VIGS, and transgenic plants either involve genetic modification of the plant genome or the use of attenuated plant viruses. Therefore, developing SIGS-based innovative, alternative strategies to regulate plant genes without genetic modifications is an important task in plant biotechnology.

**Figure 1:**
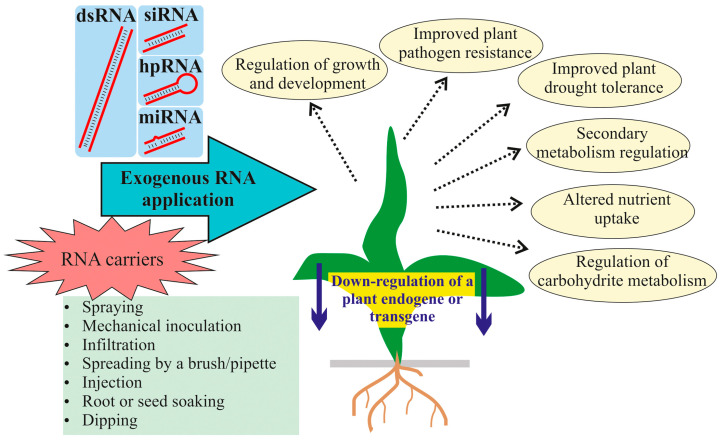
Exogenous application of dsRNA, siRNA, or hpRNA for plant gene regulation.

Exogenously added pathogen-specific dsRNAs and siRNAs in the SIGS approach are known to significantly enhance plant resistance against viral, fungal, and insect attacks after plant exogenous surface treatments [1,5,30,31,32,33]. Studies have shown that once these RNAs penetrate the plant tissue, they are processed internally by the plant and/or internalized by pests, leading to the silencing of the targeted vital genes of the invading pathogen and improved plant resistance. However, recent studies have also demonstrated that synthetic dsRNAs, siRNAs, hpRNAs, and miRNAs, when applied externally, can silence target genes within the plant genome, including both transgenes and endogenous plant genes (Figure 1; Table 1 and Table 2). These treatments can be delivered through various methods such as foliar spray, root soak, infiltration, and more. The available studies showed that the exogenous RNA treatments have led to various observable biochemical and phenotypic changes, indicating wide applicability of SIGS/exo-RNAi in plant biotechnology. For instance, the exogenous application of gene-specific dsRNAs has resulted in modifications to flower morphology [34] and increased drought tolerance [35]; siRNAs—in altered ethylene metabolism [36] and anthocyanin production [37]; miRNAs—in inhibition of primary root development [38]. Other studies are also analyzed in this review.

The systemic spread of the exogenously applied RNAs by different delivery methods throughout plant tissues has been documented [37,38,39,40,41,42,43]. There is also evidence that the exogenous dsRNAs have been subsequently processed into siRNAs by the plant RNAi machinery in the plant tissues, indicating RNAi induction [38,41,43,44,45,46,47]. Taken together, the available studies revealed that dsRNAs, siRNAs, hpRNAs, and miRNAs applied to plant surfaces can silence genes involved in a variety of pathways and biochemical processes. The studies also confirmed that exogenous RNAs can circulate within the plant vascular system and plant cells, demonstrating the effectiveness of this method.

This review provides a comprehensive summary of the current knowledge on the use of external dsRNAs, siRNAs, hpRNAs, or miRNAs for silencing of endogenous plant genes. We also analyze and discuss studies showing the ability of external RNAs to silence transgenes in plants, which is important to the understanding of exoRNAi. Furthermore, this review also discusses available information on the recognition, uptake, transport, and mechanism of action of exogenous RNAs. This opens up new avenues for developing innovative strategies in agricultural biotechnology and plant gene functional studies.

**Table 1:** External application of RNAs for suppression of plant transgenes.

Target	RNA Treatment	RNA Amount	RNA Application	Accessory Carrier/Surfactant	Plant Host for Treatment	Effect	Effect Assessment	Reference
*YFP* transgene	In vitro-synthesized short siRNA (21 bp) in a complex with a carrier peptide	100 µL of the RNA-peptide complex (20 pmol siRNA)	Infiltration	Carrier peptide (KH)9-Bp100	*YFP*-transgenic *Arabidopsis thaliana* and poplar *Populus tremula × tremuloides* (fully expanded leaves)	- Suppression of YFP protein level and fluorescence	Assessed 1, 3, 6, 9, 12, 24, and 36 hpt	Numata et al. (2014) [48]
*GFP* transgene	In vitro-synthesized siRNAs (21, 22, and 24 nt)	100 µL of aqueous siRNA solutions (10 µM)	High-pressure spraying and simple spraying	With or without Silwet L-77 surfactant	*GFP*-transgenic tobacco *Nicotiana benthamiana* (leaves and buds)	- Local and systemic GFP fluorescence suppression - Systemic silencing after spraying of 22 bp siRNAs	Assessed 2 and 20 dpt	Dalakouras et al. (2016) [49]
*GUS* transgene	Total RNA from dsRNA-expressing *Escherichia coli* HT115 (~504 bp)	100 µg of dsRNA with or without LDH	Spraying	LDH clay nanosheets or BioClay	*GUS-*transgenic *A. thaliana* (5-day-old seedlings)	Reduction in GUS activity	Assessed 7 dpt	Mitter et al. (2017) [50]
*EGFP* and *NPTII* transgenes	In vitro-synthesized dsRNAs (EGFP 720 bp; NPTII 599 bp)	0.35 µg/µL (100 µL	Spreading with brushes	-	*EGFP*- and *NPTII*-transgenic *A. thaliana* (4-week-old rosettes)	- Sequence-specific suppression of *EGFP* and *NPTII* mRNA; - Suppression of EGFP protein and fluorescence levels - Induction of *EGFP* and *NPTII* DNA methylation	Assessed 1, 7, and 14 dpt	Dubrovina et al. (2019); Kiselev et al. (2022) [44,51]
*EGFP* transgene	In vitro-synthesized siRNAs (*EGFP* 21 bp) linked to DNA nanostructures	100 μL of siRNA (100 nM)	Infiltration	siRNA-linked DNA nanostructures (3D tetrahedron, 1D monomer, 1D nanostring)	*mGFP5*-transgenic tobacco *N. benthamiana* (4-week-old plants)	- siRNA-linked 3D DNA nanostructures show *EGFP* silencing at both the mRNA and protein - siRNA-linked to 1D DNA nanostructures shows gene silencing at the protein level, but increased mRNA levels	Assessed 12 and 36 hpt or 3 and 7 dpt	Zhang et al. (2019) [52]
*GFP* transgene	In vitro-synthesized *GFP-*dsRNAs (322 and 139 bp)	200 μL of dsRNA (10, 20, 200, and 240 ng/μL)	High-pressure spraying	-	*GFP*-transgenic *Nb-16C N. benthamiana* (10–12 cm tall plants)	- No effect on GFP fluorescence; - Exogenous dsRNAs were not processed into specific siRNAs.	Assessed for 21 dpt Assessed 5 dpt	Uslu et al. (2020) [53]
*GFP* transgene	Synthetic siRNAs (22 bp)	200 μL of aqueous siRNA solution at 1 μM concentration	High-pressure spraying	-	25-day-old *GFP-*transgenic *N. benthamiana* Nb-16C plants	- Lowered GFP fluorescence - siRNA targeting the 5′ *GFP* and middle regions were more efficient when compared with the siRNAs targeting the 3′ *GFP* region	Assessed 6, 18, and 35 dpt	Uslu et al. (2022) [54]
*NPTII* transgene	In vitro-synthesized *NPTII*-siRNAs (*NPTII* 21 bp) methylated and non-methylated at 3′ ends Heterogeneous *NPTII*-siRNA mix (digestion of the *NPTII*-dsRNA)	50 pmol/μL (100 µL per plant)	Soft brushes	-	*NPTII*-transgenic *A. thaliana* (4-week-old rosettes)	- Suppression of *NPTII* mRNA levels; - A higher effect was observed for *NPTII-*siRNAs methylated at 3′ ends - Induction of *NPTII* DNA methylation	Assessed 1 and 7 dpt	Dubrovina et al. (2020) [45]
*EGFP* transgene	In vitro-synthesized dsRNAs (*EGFP* 500 bp)	1, 2, 4, or 8 μg of dsRNA per 1-week-old plant (5, 10, 20, or 40 ng/μL in 2 mL of water)	Spraying and dipping	-	*EGFP*-transgenic *A. thaliana DR5-EGFP* line (1-week-old seedlings)	Suppression of EGFP-induced fluorescence as well as *EGFP* mRNA levels	- Assessed 1, 2, 4, and 6 dpt	Park et al. (2022) [55]
*GFP* transgene	Synthetic siRNAs (22 bp) in a complex with MSNs	100 μL of the MSN-siRNA (siRNAs 10 μg mL^−1^)	Spraying Infiltration	MSNs and 0.03% Tween 20	*GFP*-transgenic *N. benthamiana* 16 C line (4–6-week-old plants)	- Reduction in *GFP* mRNA expression levels - Lowered GFP protein level and fluorescence - Reduction in *GFP* mRNA expression levels	- Assessed 1, 3, and 5 dpt - Long-term effect assessed 4, 7, 11, and 13 dpt	Cai et al. (2024) [56]
*GFP* transgene	*GFP*-RNA nanoparticles: triangle (474 nt), square (630 nt), pentagon (786 nt), and hexagon (942 nt) extracted from *E. coli HT115* GFP-dsRNA	100 ng µL^−1^	Spraying	-	*GFP*-transgenic *A. thaliana* (2-week-old plants)	- Suppression of GFP fluorescence and mRNA - RNA squares had the highest RNAi efficiency, followed by RNA triangles	Assessed 1, 4, and 7 dpt	Zhao et al. (2024) [57]
*GFP* transgene	*GFP*-dsRNA (185 bp) with CPP	500 ng of gfp-dsRNA and 5000 ng of CPP6	Infiltration	Cationic poly-aspartic acid-derived polymer (CPP6)	*GFP*-transgenic *A. thaliana* (3-week-old plants)	- Suppression of *GFP* mRNA, GFP protein, and fluorescence levels	Assessed 1, 24, and 48 hpt	Pal et al. (2024) [58]

**Table 2:** External application of RNAs for suppression of plant endogenous genes.

Plant Gene Target	RNA Treatment	RNA Amount	RNA Application	RNA Carrier	Plant Material for Treatment	Effect Assessment	Effect Assessment	Reference
*EPSPS* gene of 5-enolpyruvylshikimate-3-phosphate synthase in Palmer amaranth	In vitro-synthesized short dsRNAs (24 bp); long dsRNAs (200–250 bp)	10 µL of dsRNA on each of four leaves per plant (0.024–0.8 nM)	Leaves pre-treatment by carborundum solution or surfactant solution	-	Palmer amaranth (glyphosate-tolerant)	- Suppressed *EPSPS* transcript and protein levels - Improved glyphosate efficacy	At least for 48–72 hpt	Sammons et al. (2011) [59]
Chalcone synthase *CHS* gene	In vitro-synthesized short dsRNA (21 bp) in a complex with a carrier peptide	100 µL of protein carrier in a complex with the siRNA (6 pmol)	Infiltration	Carrier peptide (KH)9-Bp100	*Arabidopsis thaliana*	- Local loss of anthocyanin pigmentation	Assessed 2 dpt	Numata et al. (2014) [48]
*SHOOT MERISTEMLESS (STM)* and *WEREWOLF (WER)* transcription factor genes	A mixture of cationic fluorescent nanoparticles G2 and in vitro-synthesized dsRNA (*STM* 450 bp; *WER* 550 bp)	G2 nanoparticles/dsRNA complexes 2: 1 (1 mg dsRNA once per 24 h) for 3–5 days	By pipette	Cationic fluorescent nanoparticles G2	The root tip of a 10- day-old seedling of wild-type *A. thaliana*	- Suppressed *STM* and *WER* transcripts - Retarded growth and reduced meristem size; - Fluorescence observed throughout the root system (24 hpt)	At least for 5–7 dpt	Jiang et al. (2014) [60]
*DhMYB1* transcription factor gene of *Dendrobium* hybrida	Crude extract of *E. coli* HT115 containing *DhMYB1* dsRNA (430 bp)	50 µL of crude bacterial extract (2 µg/µL at 5-day intervals)	Mechanical inoculation onto a flower bud	-	Flower buds of hybrid orchid, *Dendrobium hybrida* (*D. bobby messina* × *D. chao phraya*)	- Suppressed *DhMYB1*expression - Changed phenotype of floral cells (22, 25, and 29 dpt)	At least for 29 dpt	Lau et al. (2015) [34]
*Mob1A* and *WRKY23* transcription factor genes in *A. thaliana*, *Actin* gene in rice	In vitro-synthesized dsRNAs (*Mob1A* 554 bp; *WRKY23* 562 bp)	Arabidopsis and rice seeds or seedlings soaked in 0.2 or 1 mL dsRNA (1.0 mg/mL)	Root soaking	-	Arabidopsis, rice	- Suppression of *Mob1A* and *WRKY23* - Repressed root growth and seed germination - Plants could not bolt or flower - Suppression of *Actin* - Repressed root growth	Assessed 1 dpt and 5 dpt	Li et al. (2015) [61]
*STP1* and *STP2* sugar transporter genes in tomato	In vitro-synthesized dsRNAs *STP1* and *STP2* dsRNA	300 µL of 10 ng/µL dsRNA solution per 10 germinated seeds	Seed soaking	-	Tomato *Solanum lycopersicum* seeds on the first day post-radicle emergence	- Downregulation of tomato *STP1* and *STP2* genes; - Reductions of glucose and fructose, but not xylose, in root exudate	Assessed 1 dpt	Warnock et al. (2016) [62]
The S-gene *LBDIf7* transcription factor gene in grapevine	In vitro-synthesized dsRNAs (*VviLBDIf7* 412 bp)	100 μg/plant of dsRNA in 1 mL of water	Spraying		6-year-old *Vitis vinifera* cv. Pinot noir	- Decreased *VviLBDIf7* gene expression - Reduced *Plasmopara viticola* infection and sporulation	At least for 7 dpt	Marcianò et al. (2021) [63]
*CHS*, *MYBL2*, and *ANAC032* transcription factor genes in *A. thaliana*	- In vitro-synthesized dsRNAs (736 bp for *CHS*; 588 bp for *MYBL*; 762 bp for *ANAC032*) - In vitro-synthesized siRNA (21 bp for *CHS*)	0.35 µg/µL (100 μL per plant) 50 pmol/µL (100 μL per plant)	Individual soft brushes (natural pony hair)		Four-week-old rosettes of *A. thaliana*	- Decreased anthocyanin levels after *AtCHS*-dsRNA and *AtCHS*-siRNA application - Increased anthocyanin levels and *AtCHS* expression after *AtMYBL-*dsRNA and *AtANAC032-*dsRNA	Assessed on day 2 and 7 dpt	Kiselev et al. (2021) [37]
The E2 conjugase *PHO2* gene in *A. thaliana* *SPL9* transcription factor gene in *A. thaliana*	- Total RNA, extracted from wild-type (WT) plants or from plants overexpressing either miR399 or miR156 Synthetic ds-miR399 and ds-miR156	0.01 and 1 μg in 2 mL of nutrient medium - 0.2 μM synthetic ds-miR156	Seedling soaking (50 seedlings per well)	-	*A. thaliana* (6-day-old or 8-day-old seedlings)	- Silencing of target genes *PHO2* and *SPL9* - Inhibition of primary root development - Exogenous miRNAs are translocated by the xylematic route - Exogenous miRNA-triggered RNAi requires AGO1 and RDR6	Assessed after 24 h of incubation Assessed 5 dpt	Betti et al. (2021) [38]
Phytoene desaturase *PDS* gene in citrus	In vitro-synthesized *PDS*-dsRNA (391 bp)	500 ng µL^−1^, 20 µL per leaf	Foliar application to laser-treated leaves	Laser light for leaf microperforation	*Citrus macrophylla* (leaves of 12-month-old plants)	Decreased expression of the *PDS* gene; Leaf photobleaching phenotype	Assessed 3 dpt	Killiny et al. (2021) [64]
*IAA9* and *AGL6* transcription factor genes in tomato	In vitro-synthesized dsRNAs (*SlIAA9* 717 bp; *SlAGL6* 702 bp) coupled with LDH nanoparticles	50 µL of dsRNA-LDHs (5 µg:1 mg; 1:200 *w*/*w*) or dsRNA alone	Pedicel injection	LDHs nanoparticles	Tomato *S. lycopersicum* cv UC82	- Decreased expression of *SlIAA9* and *SlAGL6* - Increase in ovary weight - RNAi was induced by the processing of injected dsRNA to 21–24 siRNAs	Assessed 5 dpt Assessed 15 dpt Assessed 5 dpt	Molesini et al. (2022) [47]
A putative glutathione S-transferase *GST40* gene in grapevine	In vitro-synthesized dsRNAs (*VvGST40* 688 bp)	50 µg of dsRNA per plant	High-pressure spraying (10 bar) 7 and 4 days before the drought	-	1-year-old *V. vinifera* cv Chardonnay	- Decreased *VvGST40* gene expression - Increased resilience to severe drought	Assessed 18 dpt	Nerva et al. (2022) [35]
Isoamylase genes *ISA1*, *ISA2*, and *ISA3* in potato	In vitro-synthesized dsRNAs (250 bp)	No data	Spraying (every 2 weeks, for a total of 6 sprays over a 15-week growth period)	lmPEI nanoparticles	Leaves of potato *Solanum tuberosum* L. cv. ’Desiree’	- Decreased expression of *ISA1* and *ISA2* genes in leaves and *ISA3* gene in tubers; - Reduced starch granule size and increased sucrose content - Early sprouting phenotype	Assessed 2, 4, 6, 8, and 10 weeks after treatment) Assessed during 120 days of cultivation	Simon et al. (2023) [65]
- The polyphenol oxidase *PPO* and the phenylalanine ammonia-lyase *PAL2* potato genes - *MYB12* transcription factor gene in potato	dsRNA-*PPO* and dsRNA-*PALX* dsRNA-*MYB12* (500 bp all dsRNAs)	20 μL of dsRNA (0.1 g/L)	Spraying	-	Fresh-cut potato slices of *S. tuberosum*	- Decreased expression of *StPPO*, *StPAL2*, and *StMYB12* genes - reduced activities of PPO and PAL - Decrease in fresh-cut potato browning	Assessed 12, 24, 48, 72, and 120 h dpt	Chen et al. (2023) [66]
*SlMYBATV1*, *SlMYB32*, *SlMYB76*, and *SlTRY* transcription factor genes in tomato	In vitro-synthesized dsRNAs (599 bp for *SlMYBATV1*; 500 bp for *SlMYB32*; 386 bp for *SlMYB76*; 285 bp for SlTRY)	70 µg of the dsRNA diluted in 400 µL of water	Spraying	-	Four-week-old tomato *S. lycopersicum*	- Downregulated mRNAs of the *SlMYBATV1*, *SlMYB32*, *SlMYB76*, and *SlTRY* genes - Upregulated expression of anthocyanin biosynthesis genes - Enhanced anthocyanin content in leaves	Assessed at 7 dpt	Suprun et al. (2023) [67]
- Flowering locus *FT* and phytochrome interacting factor 4 *PIF4* genes in *A. thaliana* Phytoene desaturase *PDS* gene in rice; *ZIP23* transcription factor gene in rice The RING-finger containing E3 ligase *SDIR1* gene in *A. thaliana* and rice Sugar transporter *SWEET14* gene in rice	ft-dsRNA-CPP6 (219 bp); pif4-dsRNA-CPP (210 bp) pds-dsRNA-CPP6 (481 bp); zip23-dsRNA-CPP6 sdir1-dsRNA-CPP6 (133 bp) for *A. thaliana*; sdir1-dsRNA-CPP6 (179 bp) for rice; sweet14-dsRNA-CPP6 (189 bp) for rice	125 ng/plant mixed with CPP6 in a 1:10 ratio 2 μg/seedling of pds-dsRNA-CPP6; 150 ng/seedling of zip23-dsRNA-CPP6 250 ng of dsRNAs per leaf 250 ng of dsRNAs per leaf	Foliar spray Root uptake (3-day-old seedlings transferred to tubes with dsRNA solution) Spraying Spraying	Cationic poly-aspartic acid-derived polymer (CPP6)	Three-week-old *A. thaliana* plants Three-day-old seedlings of *O. sativa* Three-week-old *A. thaliana* plants 45-days-old *O. sativa* plants 45-days-old *O. sativa* plants	- Decreased expression of *FT* and *PIF4* genes - Delayed flowering - Enhanced biomass - Decreased seedling height associated with dwarf and albino plant phenotypes - *PDS* transcript levels did not show any reduction compared to control plants. - Decreased expression of the *AtSDIR1* gene; - Improved resistance against *Pseudomonas syringae* pv. tomato - Decreased expression of the *OsSDIR1* gene; - Improved resistance against *Xanthomonas oryzae* pv. *oryzae* - AGO and DICER expression increased - Decreased expression of *OsSWEET14* gene; - Improved resistance against *Xanthomonas oryzae* pv. *oryzae* - Prolonged survival of bacteria	Assessed 48 hpt (the number of bolts); 10 dpt (bolting length and the number of leaves); 2, 4, 6, 8, and 10 dpt (leaves collected); 10 dpt (flowers collected) Assessed 48 hpt (gene expression) and 10 dpt (seedling height) Assessed 48 hpt (gene expression, shoot, and root length) Assessed 3 dpt Assessed at 2, 4, 6, and 10 dpt Assessed 10 dpt Assessed at 2, 4, 6, and 10 dpt Assessed 10 dpt Assessed until 60 dpt	Pal et al. (2024) [58]
The tobacco genes of magnesium chelatase *ChlH*, phytoene desaturase *PDS*, the chloroplast protein *HHL1*, and thylakoid membrane-bound protease *FtsH2.* A disease-resistant R protein, *ROQ*, and an *SOS* protein gene	Synthetic siRNAs (19–25 bp) in a complex with mesoporous silica nanoparticles (MSNs)	100 μL of the MSN-siRNA solution with 0.03% Tween 20 (siRNAs 10 μg mL^−1^)	Infiltration spraying	MSNs and 0.03% Tween 20	4–6-week-old *N. benthamiana* Plants	- Reduction in *PDS*, *ChlH*, *HHL1* and *FtsH2* mRNA levels - Photobleaching phenotype (white and yellow leaf spots) - Reduction in *ROQ* and a *SOS* mRNA ex- pression levels	Assessed 1, 3, and 5 dpt	Cai et al. (2024) [56]
*CTR4sv3* protein kinase gene in tomato	Synthetic CTR4sv3-siRNA (21 bp) designed based on the interaction site between miR1917 and CTR4sv3	10 pmol/µL and 100 pmol/µL of a siRNA Fruit injection—5 µL of a solution with 2000 pmol (400 pmol/µL) of each siRNA	- Seed soaking - Fruit injection (into green-mature tomato fruits)	-	*S. lycopersicum* cv Micro-Tom (7-d-old seedlings and mature-green fruits)	- Reduction in *CTR4sv3* mRNA; - Triple response to ethylene phenotype - Increase in the ethylene biosynthesis gene *ACO1* - Reduction in *CTR4sv3* mRNA levels - Increase in ACO1 - No noticeable changes in the fruit phenotype	Assessed 7 dpt (seedlings) Assessed 72 h after injection (fruits)	Cedillo-Jimenez et al. (2024) [36]
*MYB2* transcription factor gene from ginseng *GUT* gene from oil-seed camellia	RNA nanoparticles of square shape based on MYB2-siRNAs and *GUT*-siRNAs. The RNA NPs were synthesized and extracted from *E. coli.*	100 ng µL^−1^ of the RNA Nanoparticles	Spraying	-	Individual leaves of *Panax notoginsen*, *Camellia oleifera*	- Inhibition of *PnMYB2* and *CoGUT* expression; - RNA squares had the highest RNAi efficiency, followed by RNA triangles	Assessed at 5 and 10 dpt	Zhao et al. (2024) [57]
*CPC, MYBL2,* and *ANAC032* transcription factor genes; *CBP60g* calmodulin-binding protein gene; and *AtBAN*, anthocyanidin reductase gene in *A. thaliana*	In vitro-synthesized dsRNAs applied individually or in mixtures (218 bp for *CPC*; 588 bp for *MYBL2*; 762 bp for *ANAC032*; 724 bp for ds*CBP60g*; 486 bp for ds*BAN*)	0.35 µg of a dsRNA in 100 μL of water per plant (0.35 µg/µL) Five dsRNAs in mixtures (50 µg, 100 µg, or 150 µg of total dsRNAs equally mixed together	Individual soft brushes (natural pony hair)	-	Four-week-old rosettes of *A. thaliana*	- Significant downregulation of all five target genes - Enhanced expression of *CHS* gene - Increased anthocyanin content; - Application of the five dsRNAs in mixtures was more efficient than individual dsRNAs	Assessed 2 and 7 dpt	Kiselev et al. (2024) [68]

## 2. Silencing Transgenes in Plants Through the Application of Exogenous RNAs

Transgenes in plants are considered a valuable model for silencing gene targets by exogenous RNAs. Compared to the silencing of plant endogenes, transgenes are more sensitive to silencing, produce distinct silencing effects, and are less likely to have secondary effects [69,70,71]. Furthermore, this increased sensitivity simplifies experimental design and interpretation, reducing the risk of confounding effects from the complex interplay of endogenous gene interactions. Consequently, transgenes are useful for initial studies of exogenously induced silencing. Exploring the optimal conditions for transgene silencing through exogenous plant RNA treatments is a reasonable step before tackling the complexities of silencing plant endogenes.

Several studies have investigated the feasibility and efficacy of silencing plant transgenes by applying transgene-specific dsRNAs, siRNAs, or hpRNAs to plant surfaces (Table 1). Common transgenes such as green fluorescent protein (*GFP*), yellow fluorescent protein (*YFP*), β-glucuronidase (*GUS*), and neomycin phosphotransferase II (*NPTII*) have been targeted by this approach, resulting in suppression of transgene mRNA, protein, and observable phenotypes (e.g., reduced fluorescence in *GFP*-expressing plants (Table 1)). In these studies, exogenous RNAs were applied by approaches such as spraying, infiltration, dipping, and spreading with soft brushes. This silencing has been shown to be sequence-specific, only targeting specific transgenes and demonstrating the precision in exoRNAi induction [51]. Furthermore, experiments employing long DNA mimicking dsRNA and short DNA oligonucleotides mimicking siRNA did not show a substantial silencing effect on plant transgenes. This emphasizes the importance of RNA rather than DNA in triggering the exogenously induced silencing mechanism in plants [51]. The specificity further supports the potential of externally applied RNAs as a reliable tool for targeted plant gene regulation.

Several studies have highlighted the difficulties of using naked dsRNA and siRNA for silencing transgenes. For example, simple spraying, infiltrating, or injecting naked siRNAs targeting the *GFP* transgene in tobacco and Arabidopsis had a low effect on transgene fluorescence and failed to elicit significant silencing [49]. This suggests the need for improved delivery methods. This issue was addressed by applying high-pressure sprays to tobacco, apple, and grapevine [49,72] or by utilizing different carriers for tobacco or *A. thaliana*, such as carrier peptides (CPPs) [48,58], the layered double hydroxide (LDH) clay nanosheets or BioClay [50], DNA nanostructures [52], and mesoporous silica nanoparticles (MSNs) [56]. The CPPs, DNA nanostructures, and MSNs proved to be more effective than naked dsRNAs in delivering transgene-encoding siRNAs in plants, resulting in considerably reduced YFP and GFP fluorescence, protein amounts, and mRNA levels. However, the LDH clay nanosheets were effective at reducing *GUS* activity at approximately the same level as naked dsRNA [50]. The challenges and lower efficiencies of direct dsRNA or siRNA application could be attributed to several factors, such as RNA degradation, low cellular uptake, or inefficient RNA transport. This highlights the necessity for addressing these issues through various strategies, including the improvement of delivery systems, RNA chemical modifications, and optimizing RNA design. On the other side, several further studies have shown that treating plants externally with dsRNA or siRNA without additional instruments or carriers can significantly decrease transgene expression [44,45,50,51,55,57].

The efficiency with which exogenous RNAs are penetrated, processed, and acted upon may be affected by certain experimental or natural factors, which can constrain or enhance the ultimate silencing effect. However, there is a lack of studies on the effect of physiological conditions at the time of plant treatments on the efficiency of exogenously induced silencing in plants. A study has shown the profound impact of the physiological conditions at the time of dsRNA application on *NPTII* transgene silencing in *A. thaliana* [73]. It has been shown that the time of day, plant maturity, and soil moisture can profoundly affect the efficacy of exogenously induced silencing in *A. thaliana* [73]. The application of dsRNA at later periods of the day, particularly at night, resulted in significantly higher levels of transgene expression than applying it during the day. This suggests a potential link between plant circadian rhythms and the cellular machinery responsible for exoRNAi induction. Considering dsRNA delivery methods, brush spreading, spraying, and pipetting, particularly when applied to both adaxial (upper) and abaxial (lower) leaf surfaces, showed far greater efficacy compared to methods such as infiltration or inoculation [73].

In addition to achieving transgene silencing induced by exogenous RNAs, it is necessary to study the mechanism of this phenomenon and prove that RNAi actually occurs. 22-nt siRNAs have been shown to be the most potent inducers of local and systemic silencing when compared with 21-nt and 24-nt siRNAs [49]. After spraying naked dsRNAs under high pressure into transgenic tobacco, Uslu et al. [53] used small RNA sequencing (sRNA-seq) to analyze whether exogenous *GFP*-dsRNA was processed into siRNAs. The results showed minimal processing of the exogenous *GFP*-dsRNA into siRNAs, and GFP fluorescence was reduced only locally to a limited level. The data indicated that exogenous *GFP*-dsRNA did not trigger the RNAi-mediated silencing of the *GFP* reporter gene in the treated tobacco leaves. However, in the following work, using sRNA-seq analysis, the authors revealed that the exogenous application of 22-nt *GFP*-siRNAs to tobacco has induced local silencing, which led to silencing amplification via transitivity [54]. The findings also suggested that transitivity and systemic silencing were tightly connected. A study on the effect of exogenous *NPTII*-coding dsRNAs on *NPTII*-transgene silencing in *A. thaliana* showed effective processing of the dsRNAs into small RNAs (sRNAs) [46]. These findings are also supported by earlier stem-loop PCR data on *EGFP* siRNA detection [44,45].

Taken together, the described results demonstrate that using exogenous RNAs to silence plant transgenes can be successful despite existing challenges. This suggests that the approach can be developed and applied to silence endogenous plant genes. By overcoming the limitations in delivery and optimizing application parameters, this technology could become a powerful tool, enabling precise control over gene expression in plants.

## 3. Silencing Plant Endogenes by the Application of Exogenous RNAs

Early evidence that exogenous dsRNA and siRNA can be used to regulate the expression of endogenous genes in plants was provided by a patent and several other studies [34,48,59,60,61,62]. For example, a patent by Sammons et al. (2011) [59] demonstrated the feasibility of using dsRNA and/or siRNA to reduce the levels of mRNA of herbicide-resistance genes in plants via the foliar RNA application. Based on a review of the existing literature, it was found that over time, further research emerged indicating that exogenous dsRNA, siRNA, and even miRNA can lead to a decrease in expression of plant endogenous genes. As summarized in Table 2, a number of research efforts have demonstrated that the application of RNAs targeting specific plant genes can reduce the mRNA and protein levels of these genes, leading to anticipated changes in plant morphology, physiology, and biochemistry. The exogenous RNA treatments can regulate different processes, including plant growth and development [34,38,47], hormonal signaling [36], pathogen resistance [58,63], abiotic stress tolerance [35], biosynthesis of plant secondary metabolites [58,66,67,74], and plant carbohydrate metabolism [62,65].

Considering morphological, developmental, and growth changes, the exogenous application of dsRNA and siRNA has demonstrated significant effects across various plant species. The application of the technique has impacted plant growth, development, and phenotype (Figure 1). Lau et al. [34] provided early evidence of the efficacy of this technology, demonstrating that the direct application of a crude bacterial extract containing *MYB1*-dsRNAs to the flower buds of the orchid, *Dendrobium hybrid*, suppressed the expression of the target *DhMyb1* transcription factor gene. This resulted in a striking phenotypic alteration—a change in epidermal cell morphology from conical to flattened [34]. Other early evidence includes the application of dsRNA targeting the genes of transcription factors *Mob1A* and *WRKY23* in *A. thaliana*, regulating organ growth and tissue patterning, which has led to repressed root growth, seed germination, and flowering [61]. Simultaneously, the application of dsRNA targeting the rice *Actin* gene, playing a crucial role in plant growth and development, has led to repressed root growth, seed germination, and flowering [61]. It should be noted that these studies have applied naked dsRNA to the plant surfaces by root and seed soaking, and mechanical inoculation. Jiang et al. [60] treated *A. thaliana* with target-specific dsRNA in a mixture with cationic fluorescent nanoparticles G2 and revealed that SHOOT MERISTEMLESS (*STM*) and WEREWOLF (*WER*) transcription factor genes were downregulated, leading to retarded growth and reduced meristem size. *STM* is known as an important regulator of meristem formation and maintenance, while *WER* is required for non-hair cell specification in plant roots. These results underscore the potential for broad-spectrum manipulation of developmental processes in plants using exogenous RNAi.

Subsequent studies expanded upon the initial success. Molesini et al. [47] showed that applying both naked dsRNAs and dsRNAs linked to LDHs via injection into the pedicels of tomato, *Solanum lycopersicum*, suppressed the expression of the ovary growth transcription repressor genes *Indole Acetic Acid 9* (*IAA9*) and *AGAMOUS-LIKE6* (*AGL6*) [47]. The outcome was a significant increase in ovary weight, which demonstrated the potential to increase fruit yield through targeted exoRNAi. According to Cedillo-Jimenez et al. [36], exogenous application of naked siRNAs to *S. lycopersicum* via seed soaking and injection into mature green fruits suppressed the expression of the target *CTR4sv3* protein kinase gene. This gene is known as an important suppressor of ethylene signaling in plants. The successful silencing of *CTR4sv3* induced the classic “triple response” to ethylene phenotypic traits, which is characterized by reduced root and hypocotyl length coupled with the noticeable development of hooks [36]. This study emphasizes the ability of RNAi to manipulate hormonal pathways and influence various aspects of plant growth, such as the stress response, flowering time, or fruit ripening. Furthermore, Betti et al. [38] investigated the capability of exogenously applied miRNAs to silence plant genes and influence developmental processes and phosphate signaling in *A. thaliana*. Exogenous application of miR156, which targets the gene of transcription factor *SPL9* (involved in juvenile-to-adult transition and other processes), inhibited primary root development in *A. thaliana*, validating the predicted function of this miRNA [38]. Similarly, applying synthetic miR399 targeting a ubiquitin-conjugating E2 enzyme gene, *PHO2* (involved in phosphate signaling), demonstrated the potential of exoRNAi to regulate the nutrient responses in plants [38]. These results demonstrate the applicability of exogenous miRNAs for the regulation of nutrient uptake and growth in plants.

Several studies have highlighted the efficacy of exoRNAi in improving plant resistance to microbial pathogens and parasitic nematodes. It has been shown that targeting the *LBDIf7*, a transcription factor in *Vitis vinifera,* with dsRNA resulted in a significant reduction in *Plasmopara viticola* infection and sporulation [63]. *LBDIf7*, acting as a repressor of plant immune responses, was effectively silenced, leading to enhanced defense against this devastating pathogen. Similarly, Pal et al. [58] targeted the sugar transporter *SWEET14* gene and the RING-finger containing E3 ligase *SDIR1* gene in *A. thaliana* and rice, both of which negatively regulate plant defenses against bacterial pathogens. The application of dsRNAs resulted in decreased bacterial growth and lesion length in both species, demonstrating improved resistance to *Pseudomonas syringae* pv. tomato in *A. thaliana* and *Xanthomonas oryzae* pv. oryzae in rice. The remarkable persistence of the bacterial resistance in rice, extending up to 30 days post-treatment, highlights the long-lasting impact of exoRNAi. One of the earliest studies by Warnock et al. [62] demonstrated that targeting the sugar transporter genes *STP1* and *STP2* in tomato by tomato seed soaking in dsRNA resulted in reduced levels of glucose and fructose, but not xylose, in collected root exudate. This, in turn, led to reduced infectivity of the plant-parasitic nematode *Meloidogyne incognita*. The applicability of exoRNAi extends beyond biotic stress responses. For instance, Nerva et al. [35] found that applying dsRNA to target the putative grapevine glutathione S-transferase *GST40* gene in grapevine increased plant resistance to severe drought. Thus, despite glutathione S-transferases acting as crucial detoxifying enzymes, the data by Nerva et al. [35] confirmed the positive role of this *GST* in plant drought tolerance.

Recent studies have also provided evidence of the possibility of regulating plant responses to stress through the exogenous application of siRNAs. Cai et al. [56] documented the downregulation of both disease resistance (a disease resistance R protein *ROQ*) and abiotic stress response (*SOS* protein kinase) genes in tobacco, indicating the potential for broad-spectrum stress regulation using this delivery method. The use of MSNs enhanced the efficacy and stability of siRNA delivery, improving the overall effectiveness of the treatment. Similarly, Zhao et al. [57] demonstrated that the *MYB2* transcription factor gene from *Panax notoginseng* was downregulated by *MYB2* siRNA-based RNA nanoparticles, which had the highest gene silencing efficiency of the square-shaped nanoparticles.

Studies have demonstrated that the external application of dsRNAs and siRNAs can regulate plant secondary metabolism. For instance, these methods influenced the expression of genes associated with anthocyanin synthesis [37,67,68,74], phenolic compound accumulation [66], and carotenoid biosynthesis [58]. Anthocyanins, which arise from the phenylpropanoid biosynthetic pathway, are a group of colored secondary metabolites that contribute to the vibrant colors of various fruits, flowers, and vegetables. Anthocyanins are valued for their antioxidant and anti-inflammatory effects and their applications in the food industry and horticulture [75,76]. Foliar application of dsRNAs has been shown to downregulate anthocyanin content through the silencing of genes encoding anthocyanin biosynthetic enzymes, such as *CHS* and *ANS*, in tomato *S. lycopersicum* and *A. thaliana* [37,68]. Conversely, the exogenously induced silencing of regulatory transcription factor genes, such as *CPC*, *MYBL2*, and *ANAC032*, or the competitive enzyme gene *BAN* and the calmodulin-binding protein gene *CBP60g*, which negatively affect anthocyanin production in *A. thaliana*, has been demonstrated to enhance *CHS* gene expression. The *CHS* gene is involved in anthocyanin biosynthesis. This ultimately led to an increase in anthocyanin production, as reported by Kiselev et al. [68]. Similar results have been obtained for tomato *S. lycopersicum,* where exogenously induced silencing of *SlMYBATV1*, *SlMYB32*, *SlMYB76*, and *SlTRY* transcription factor genes, negatively regulating the biosynthesis of anthocyanins, was associated with elevated expression levels of anthocyanin biosynthesis-related genes, *SlCHS* and *SlANS*, and a corresponding rise in anthocyanin concentrations in the leaves of *S. lycopersicum* [67]. This highlights the potential of exoRNAi for manipulating plant pigment production and potentially other plant traits. Similarly, dsRNA-mediated downregulation of phenolic metabolism in potato has been observed, resulting in a suppression of enzymatic browning in fresh-cut potato products. Exogenous application of dsRNAs targeting *Solanum tuberosum* genes involved in enzymatic browning and phenolic metabolism—specifically the polyphenol oxidase *PPO* and the phenylalanine ammonia-lyase *PAL2* genes—has demonstrated efficacy in reducing both gene expression and enzymatic activity of *PPO* and *PAL2* in fresh-cut potatoes [66]. This, in turn, reduced enzymatic browning in fresh-cut potatoes.

Exogenous application of dsRNAs has been observed to induce phenotypic alterations in rice seedlings and citrus plants by targeting the phytoene desaturase *PDS* gene, encoding an important rate-limiting enzyme in carotenoid biosynthesis [58,64]. These alterations include decreased seedling height and a dwarf, albino phenotype in rice seedlings, as well as decreased expression of the *PDS* gene and a leaf photobleaching phenotype in citrus. However, despite these changes, quantitative analyses of *PDS* expression levels in rice seedlings did not reveal a significant reduction. The authors suggest that this lack of measurable gene downregulation may be attributed to the critical role of *PDS* during the early developmental stages of rice growth. Additionally, the delivery of siRNAs using MSNs against both the *PDS* gene and the magnesium chelatase (*ChlH*) gene, which is implicated in the biosynthesis of photosynthetic pigments, has produced visible phenotypic effects such as white spots and yellowing in tobacco plants [56]. Similarly, the foliar application of siRNAs targeting *HHL1* and *FtsH2* genes encoding chloroplast proteins involved in the photosystem II repair cycle has induced photobleaching phenotypes. These results collectively demonstrate the potential of exoRNAi for manipulating photosynthetic processes and chlorophyll production in plants.

Beyond photosynthetic genes, Simon et al. [65] successfully targeted starch metabolism in potato leaves using naked dsRNAs and dsRNAs coupled with lipid-modified PEI nanoparticles. This approach suppressed the expression of isoamylase genes *ISA1*, *ISA2*, and *ISA3*, resulting in smaller starch granules, elevated sucrose levels, and early sprouting. It should be noted that Warnock et al. [62] also revealed that carbohydrate metabolism can be regulated by exogenous dsRNAs, showing that dsRNA downregulated *STP1* and *STP2* sugar transporter genes in tomato, resulting in reductions of glucose and fructose, but not xylose, in collected root exudate.

In conclusion, exogenous application of RNAs, including dsRNAs, siRNAs, and even miRNAs, has demonstrated significant effects across different plant species, impacting growth, development, morphology, stress resistance, and metabolism. While impressive progress has been made in demonstrating the feasibility of silencing plant genes via surface RNA application, several challenges remain. These include optimizing RNA production and delivery methods for different plant species and tissues, understanding the mechanisms underlying exoRNAi and variable silencing efficiencies, and developing strategies to overcome potential off-target effects, which are critical for future applications.

## 4. RNA Production for Plant Exogenous Treatments

**Figure 2:**
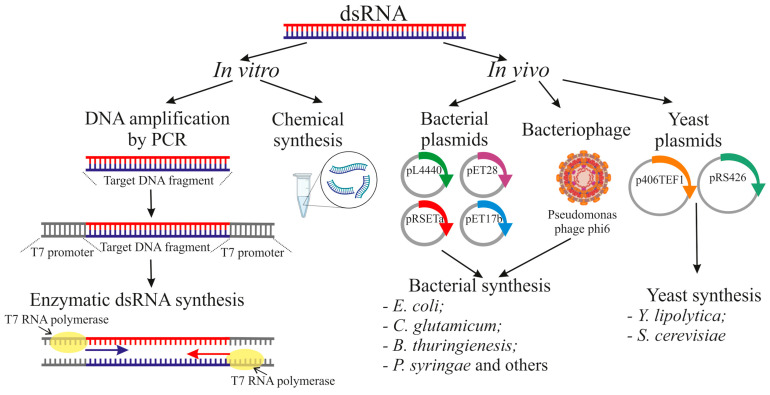
The representation of the in vitro and in vivo dsRNA production strategies for plant exogenous treatments. The strategies include enzymatic dsRNA synthesis using DNA-dependent T7 RNA polymerase, chemical synthesis, and bacterial and yeast dsRNA production systems. pL4440, pET28, pRSETa, pET17b, and phage phi6—vector systems used for dsRNA production in bacteria (*Escherichia coli*, *Corynebacterium glutamicum*, *Bacillus thuringienesis*, and *Pseudomonas syringae*). P406TEF1 and pRS426—vector systems used for dsRNA production in yeast (*Yarrowia lipolytica* and *Saccharomyces cerevisiae*).

Currently, the widespread use of exoRNAi in agriculture is constrained by the high cost and technological difficulties of large-scale production of dsRNA. At present, two fundamentally different approaches to dsRNA production for plant exogenous treatments have been developed and are actively studied: in vitro (extracellular systems) and in vivo (cellular systems) (Figure 2). Each method has unique advantages, limitations, and optimal applications.

Enzymatic synthesis in vitro, carried out using DNA-dependent RNA polymerases of bacteriophages T7, T3, or SP6, remains the gold standard for obtaining highly purified dsRNA preparations [2,77]. This process includes several key steps: first, a DNA template is created using PCR amplification of the target sequence flanked by T7 or other promoters included in the PCR primers. Then, individual RNA strands are transcribed, and finally, complementary strands are annealed to form dsRNA. Although this method provides exceptional purity (up to 99%) and allows precise control over the length and sequence of the resulting molecules, its main drawbacks—the high cost of commercial kits and limited scalability—make it impractical for agricultural applications [78]. However, most scientific studies involving the use of dsRNA rely on in vitro synthesis using commercial kits [46,47,74,79,80,81,82]. An alternative to in vitro synthesis is chemical synthesis. Although this allows for the production of highly stable, modified dsRNA molecules, it is even more expensive, particularly for long sequences [83].

Scalable and cost-effective dsRNA production may be achieved through in vivo systems utilizing genetically modified bacteria such as *Escherichia coli* [84], *Corynebacterium glutamicum* [85], *Bacillus thuringienesis* [86], and *Pseudomonas syringae* [87], as well as the yeast *Yarrowia lipolytica* [88]. The most widely studied and used strain for the production of dsRNA upon induction by isopropyl β-D-1-thiogalactopyranoside (IPTG) is *E. coli* HT115(DE3) and the plasmid pL4440. This strain has several important modifications: RNase III deficiency (which prevents dsRNA degradation), the presence of the λDE3 prophage encoding T7 RNA polymerase, and a plasmid expression system with inverted repeats [77]. To improve the efficiency of dsRNA production, other RNase III-deficient *E. coli* strains, such as M-JM109, M-JM109lacY, and BL21 (DE3), were also created [89]. This system is being optimized in several directions simultaneously: replacing the expensive and toxic IPTG inducer with cheaper and safer analogs (lactose, skim milk components), using constitutive promoters that avoid the need for induction, and creating new strains with improved characteristics [89,90]. Interestingly, deletion of the *rnc* gene (encoding RNase III) in the BL21 (DE3) strain allowed the dsRNA yield to be increased threefold (up to 4.23 μg/mL) compared to the standard L4440-HT115 (DE3) system (1.3 μg/mL) [91]. Despite the many advantages of this approach, it is important to note that the process of isolating and purifying dsRNA from bacteria is labor-intensive and also an obstacle to large-scale production. However, research aimed at simplifying the procedure for isolating dsRNA from bacteria can reduce the cost of dsRNA production and expand the application of dsRNA [92,93]. Another option for producing dsRNA includes recombinant bacterial and yeast strains that can potentially be used without the preliminary purification of the dsRNA [85,94,95,96,97]. These strains, both live and heat-inactivated, have several advantages, including reduced costs, safe status, ease of cultivation, and the ability to be stored long-term. However, this approach is still less efficient than the standard *E. coli* L4440-HT115(DE3) [89].

Alternative dsRNA production methods, such as the bacteriophage ϕ6 system, are of particular interest. Unlike traditional methods, this system uses an RNA-dependent RNA polymerase (RdRp) of bacteriophage 6 (a dsRNA virus) to directly synthesize dsRNA from a single-stranded template, ensuring high efficiency and reproducibility [98,99]. One significant advantage of this technology is the ability to produce long dsRNA molecules with high fidelity, which is especially valuable for research and therapeutic applications [99]. Successful trials of dsRNA produced by this system against tobacco mosaic virus in *N. benthamiana* have confirmed its practical applicability [87].

In summary, a comparative analysis of various dsRNA production methods reveals their respective strengths and weaknesses. While in vitro systems are ideal for research and therapeutic applications requiring high product purity, in vivo approaches show more promise for agricultural use, where cost-effectiveness and scalability are critical. Further developments in this area will likely involve creating hybrid technologies that combine the advantages of different approaches, as well as developing new platforms for the targeted delivery of dsRNA to plants. This will enable the full potential of exoRNAi for plant gene regulation or other purposes.

## 5. RNA Delivery Methods for Exogenous Plant Treatments

Studies have revealed significant challenges associated with directly applying dsRNAs or other RNAs to plant surfaces for gene regulation. Several factors may reduce the efficiency of exoRNAi or lead to its instability. First, physical barriers such as the plant cuticle and cell wall hinder the penetration of dsRNA and siRNA. Second, naked RNA molecules are unstable in the environment and degrade rapidly [100,101]. Third, the RNA uptake mechanisms are not fully understood and may vary considerably between different plant species and tissues. To overcome these limitations, researchers have explored several strategies. These strategies include optimizing different RNA delivery methods and developing RNA carriers that enhance RNA penetration and efficacy in plants. This could potentially help overcome limitations associated with traditional application methods, leading to more consistent and effective gene silencing. To successfully implement exoRNAi-based gene silencing, the initial and pivotal step involves choosing the appropriate delivery system for exogenous RNAs.

Synthetic dsRNAs, siRNAs, hpRNAs, and miRNAs have been directly applied to the plant surface by spraying, mechanical inoculation, infiltration, spreading by soft brushes, spreading by a pipette, root or seed soaking, dipping, and injections (Figure 1; Table 1 and Table 2). The most commonly used methods of treating plant surfaces with RNA to silence transgenes (Table 1) and endogenes (Table 2) were foliar spraying, soaking, spreading with a brush or pipette, and infiltration. When considering dsRNA delivery methods, spraying, brush spreading, and pipetting showed far greater efficacy compared to methods such as infiltration or inoculation [73]. This was particularly the case when applied to both adaxial (upper) and abaxial (lower) leaf surfaces. However, this investigation did not analyze the efficiency of root and seed soaking. It is possible that dsRNA is absorbed more efficiently through the root system because roots are naturally designed to absorb nutrients. Indeed, early studies have confirmed the uptake of high molecular weight RNA by plant roots [102], suggesting that the root system can play a significant role in the uptake of these molecules. Recent research advancements have confirmed effective uptake and systemic translocation of exogenous dsRNA throughout the plant after root soaking/drenching, further supporting this hypothesis [42,61,72,103].

Beyond the surface applications, other delivery approaches are being explored. Research by Dalakouras et al. [72] and colleagues showed the effective introduction of exogenous hpRNAs and siRNAs into trees as well as non-woody plants, including *Malus domestica* (apple), *Vitis vinifera* (grapevine), and *N. benthamiana* by trunk injections, petiole absorption, and soil and root drenching. Trunk injection, for example, is proving effective for delivering RNA to woody plants, allowing for systemic movement and silencing throughout the plant [72]. Soil/root drench methods deliver RNA to the root system, which can then be transported to other parts of the plant, although uptake efficiency may be variable due to factors such as soil composition and root morphology. A study by Pampolini et al. [104] also shows systemic dsRNA distribution in plant tissues after root soak and petiole absorption. Petiole absorption is a promising method for targeted delivery. In this process, the cut end of a petiole (leaf stalk) is treated with RNA, which is then drawn into the plant vasculature by capillary action. The studies indicated that this approach resulted in rapid uptake and effective systemic transport of exogenous dsRNA or hpRNA, demonstrating its potential for precise gene regulation in plants [72,104].

Recent advancements also highlight the potential of enhancing RNA uptake through physical methods. High-pressure spraying by using an airbrush has been successfully used to increase the penetration of RNA into tobacco leaf tissues and to improve the effectiveness of RNA silencing of *GFP* transgene in plants [49,72]. Furthermore, a study revealed that pre-treating citrus leaves with laser light to create microperforations improved the silencing of the target endogenous gene in the plant [64]. The laser light was used to puncture microscopic holes within the lipidized leaf surface without extensive damage to the leaf tissue.

Overall, the field of RNAi-mediated crop protection is actively pursuing enhanced delivery methods to maximize efficiency and enable broader applications in agriculture. The efficiency of exogenous RNAs in inducing plant gene silencing was promoted by applying RNA carriers. Although the literature data on the stability of dsRNAs are often contradictory, it is clear that dsRNAs can be rapidly degraded by various biotic or abiotic factors [100,101]. Exposure to sunlight, particularly UV radiation, causes rapid RNA degradation. Furthermore, dsRNA is destroyed in water primarily due to microbial activity. The diverse microbial communities inhabiting the phyllosphere (the surface of leaves) and rhizosphere (the soil surrounding plant roots) further contribute to dsRNA degradation through enzymatic activity. Many soil microbes possess RNases, thereby diminishing the effectiveness of exogenous RNAs. To overcome these challenges, various carrier systems were explored to encapsulate and protect dsRNA and siRNA, thereby enhancing their stability and improving uptake by plant tissues. These delivery systems are designed to shield dsRNA from environmental stress and enzymatic degradation. This increases RNA persistence on the plant surface, promoting penetration and ultimately improving the efficacy of RNAi-based gene regulation or pathogen control.

Significant research has been conducted regarding human RNA treatments focused on siRNA and dsRNA therapies, using carrier substances [105,106]. These approaches might also be considered for agricultural applications. Currently, the main classes of siRNA and dsRNA non-viral carriers include nanoparticles, cell-penetrating peptides (CPPs), and lipid-based vectors [107,108]. Nanoparticles include a variety of actively developed tools for delivering RNA to cells, including non-lipid organic-based nanovectors (e.g., chitosan, polyethylenimines (PEIs), dendrimers) and non-lipid inorganic-based nanovectors (MSNs, LDHs, gold nanoparticles, carbon dots) [107,108]. Apart from the carriers mentioned, the field of DNA- and RNA-based nanostructures is an emerging area for drug delivery in human medicine [109,110], as well as for potential agricultural applications involving exogenous plant treatments with RNA. DNA- and RNA-based nanostructures are engineered from DNA or RNA molecules that self-assemble into highly organized and programmable configurations, making them ideal candidates for protecting RNA and ensuring its targeted delivery. These structures can provide protection for RNA molecules and control their release, offering precise control over the timing and location of RNAi activation.

The available literature on the use of exogenous RNA to target plant genes or transgenes reveals that a number of studies have used different carriers, including CPPs [48,58] and nanoparticles such as cationic fluorescent nanoparticles G2 [60], LDHs [47,50], lmPEI nanoparticles [65], or MSNs [56] (Table 2). Furthermore, two studies report on using siRNA-based RNA nanoparticles [57] and siRNA-linked DNA-based nanostructures [52] for siRNA delivery and exoRNAi induction in plants. Several studies [48,52,56,60] have demonstrated that carriers can significantly enhance the efficacy of transgene or endogene silencing when exogenous RNAs are applied in combination with carriers or are linked to DNA-based nanostructures (see Table 1 and Table 2). However, in some cases, the effect of RNA with carriers may be comparable to that of naked RNA [47,58]. It should be noted that there are currently no studies using other carriers (e.g., chitosan, lipid-based vectors, carbon dots) for silencing endogenous plant genes. Therefore, there is a large field for further research on endogenous gene regulation in plants.

In summary, the choice of delivery method significantly impacts the efficiency and consistency of exoRNAi in plants. This includes approaches such as naked RNA application, nanoparticle-mediated delivery, or other technologies such as laser light or high-pressure spraying. Often, naked RNA shows negligible or no effect, highlighting the critical role of effective delivery and carrier systems in achieving successful plant protection. Further research focusing on optimizing carrier design, understanding plant-carrier interactions, and exploring novel delivery approaches will be instrumental in realizing the full potential of exoRNAi.

## 6. Exogenous RNA Recognition, Uptake, and Transport in Plants

Several studies utilizing confocal microscopy and other advanced techniques have demonstrated that exogenous dsRNAs, siRNAs, hpRNA, and miRNAs can indeed be absorbed by plants and subsequently transported through the plant vascular system [37,38,39,40,41,42]. However, it is recognized that several physical barriers can significantly hinder the absorption of dsRNA when applied exogenously. For example, the presence of trichomes, the cuticle, and cuticular wax on the leaf surface are important factors that influence this process and affect the wettability and hydrophobicity of leaves [101]. Reduced wettability makes it increasingly difficult for dsRNA or other RNA molecules in the foliar spray to effectively penetrate the leaf surface. Consequently, the uptake of RNA applied to the leaf surface is often limited by these barriers.

Foliar-applied dsRNAs were detected in the xylem of barley leaves, apoplast and symplast of phloem parenchyma cells, companion cells, and mesophyll cells, as well as in trichomes and stomata [39]. According to Mitter et al. [50], both naked viral dsRNA and viral dsRNA loaded on LDH were taken up into the xylem of Arabidopsis leaves. Using confocal microscopy, Dalakouras et al. [72] revealed that the uptaken and systemically transported RNA molecules were strictly confined to the xylem and apoplast. This may also explain why the hpRNAs applied were not processed into siRNAs by plant DCL endonucleases.

A study by Song et al. [111] revealed that wheat coleoptiles absorb exogenous dsRNA more effectively through wounded surfaces than healthy ones. This absorption process suggests that the dsRNA is transported through the tracheary elements in the plant. The findings indicate that the damaged areas of the plant exhibit a significantly higher capacity for dsRNA uptake, highlighting the potential for using techniques such as abrasion or high-pressure spraying to facilitate penetration of dsRNA into plant tissues. Furthermore, research by Faustinelli et al. [40] provided insights into the systemic movement of exogenously applied synthetic siRNAs in peanut plants. Their study demonstrated that these siRNAs not only spread throughout the plant but also remained stable for at least 30 days in vitro. Betti et al. [38] found that exogenous Cy3-labeled miR399 is translocated by the xylematic route. It was demonstrated that these miRNAs spread along the xylem of Arabidopsis seedlings, with no specific signal observed in the phloem. miRNAs were also found to spread from one plant to another via the same hydroponic nutrient medium.

There are several pathways through which RNA molecules can theoretically enter the plant, including the cuticle, stomata, and minor leaf surface injuries, providing multiple avenues for uptake. Nevertheless, the exact process by which externally applied RNAs are taken up remains unclear. It is hypothesized that the absorption of dsRNA through the root system should be more efficient as the roots are naturally designed to absorb nutrients. Indeed, several studies have confirmed the uptake of dsRNA by plant roots, suggesting that the root system could play a significant role in the uptake of these molecules [42,61,72,103]. The precise mechanisms by which plants absorb exogenous dsRNAs and other RNA molecules remain largely elusive. Research has provided insights into the nematode *Caenorhabditis elegans*, which utilizes a specific mechanism for the uptake of external dsRNAs that involves the systemic RNAi defective protein 2 (SID-2) transmembrane protein [112]. This protein plays a crucial role in allowing the nematode to incorporate dsRNA from the environment. For plants, several studies have shown that the uptake of naked dsRNA, siRNA, and miRNA molecules, as well as the exogenous induction of gene silencing, is feasible within plant cells [36,37,38,42,44,45,47,61,62,66,67,105]. However, whether this uptake is mediated by a carrier protein, similar to the SID-2 mechanism in *C. elegans*, remains an open question. The exogenous dsRNAs and siRNAs (Table 1 and Table 2) may be taken up and processed by the same natural pathways that allow extracellular nucleic acids from microbial pathogens, insects, or viruses to enter plant cells. Despite the growing interest in this field, the current literature offers limited insights into the specific mechanisms that facilitate the recognition, uptake, and translocation of exogenously applied nucleic acids within plant tissues.

Research has shown that extracellular RNA and DNA from pathogenic microorganisms and viruses can trigger innate immune responses in plants [113,114,115,116]. This phenomenon is crucial for plant defense, as these molecules can regulate self- and non-self-recognition processes. They are often perceived as microbe- and pathogen-associated molecular patterns (MAMPs and PAMPs), which are recognized by pattern recognition receptors (PRRs) on the plant cell surface [115]. The activation of these receptors leads to pattern-triggered immunity (PTI), a fundamental component of the plant immune system. However, data on how plants perceive and respond to pathogenesis-related DNA and RNA are scarce. To date, specific receptors that mediate the recognition and uptake of extracellular DNA and RNA have not been clearly identified in plants. Recent findings by Niehl et al. [115] have provided some clarity on this issue. Their research demonstrated that the application of virus-related dsRNA can induce PTI responses in Arabidopsis plants via a receptor known as somatic embryogenesis receptor-like kinase 1 (SERK1). Interestingly, this response occurred independently of the antiviral DCL proteins, suggesting that dsRNA-mediated PTI may involve membrane-associated processes that operate outside the traditional RNA silencing pathways. Interestingly, in a recent study, Samarskaya et al. [117] have shown that the exogenous application of synthetic viral dsRNA to potato plants induced the accumulation of both siRNAs and the PTI-related gene transcripts such as *WRKY29* (WRKY transcription factor 29; molecular marker of PTI), *RbohD* (respiratory burst oxidase homolog D), *EDS5* (enhanced disease susceptibility 5), *SERK3* (somatic embryogenesis receptor kinase 3) encoding brassinosteroid-insensitive 1-associated receptor kinase 1 (BAK1), and *PR-1b* (pathogenesis-related gene 1b). This suggests that externally applied dsRNAs may induce the PTI-related responses.

In conclusion, despite the challenges of penetration, exogenously applied dsRNAs can be absorbed by plants and subsequently transported by the plant vascular system. This is also indirectly supported by literature data on the documented effects of dsRNA and siRNA on plant gene expression in the absence of any carriers. Although the mechanisms underlying the uptake and recognition of exogenous dsRNAs and siRNAs in plants remain poorly understood, emerging research highlights their potential role in enhancing plant immunity.

## 7. Processing of Exogenous dsRNA into siRNA in Plants

Several studies have confirmed the effective processing of externally introduced dsRNAs into functional siRNAs in plants, highlighting the potential of exoRNAi as a tool for plant gene regulation. For example, research conducted by Molesini et al. [47] using sRNA-seq showed that when the dsRNAs specific for the *IAA9* and *AGL6* genes were introduced into plant ovaries in combination with LDHs, the dsRNAs were processed into gene-specific siRNAs ranging from 21 to 24 nt in length. These siRNAs were not detected when only LDHs were applied. Notably, the sRNAs were mapped to the portion of the *IAA9* and *AGL6* sequences chosen for dsRNA production, with some regions generating more sRNAs than others. Additionally, other studies have also corroborated the successful processing of dsRNA into siRNA in plants. For example, two studies utilized stem-loop PCR to detect siRNAs corresponding to the *EGFP* and *NPTII* genes in *A. thaliana* [44,45]. The research revealed that in vitro-synthesized dsRNAs targeting the coding regions of these transgenes effectively suppressed transgene transcript levels. This was accompanied by the detection of specific *EGFP*-siRNA in the treated plants using stem-loop PCR.

Nityagovsky et al. [46] expanded upon this by employing sRNA-seq to investigate the effects of foliar applications of dsRNAs that target the non-related *NPTII* gene and the endogenous *AtCHS* gene in wild-type *A. thaliana*. The results showed that there was a significant accumulation of *NPTII*- and *AtCHS*-specific sRNAs in the treated plants, whereas these were absent in the control plants that received only water. The study found that the most abundant sRNAs were 21-nt, 23-nt, and 24-nt in length, highlighting the efficacy of the dsRNA treatments in eliciting a robust RNAi response. Treating plants with *AtCHS*-dsRNAs resulted in a significant increase in the amount of 21-nt sRNAs. In contrast, the levels of 23-nt and 24-nt sRNAs decreased compared to other treatments. The findings demonstrated that the exogenous *AtCHS*-encoding dsRNA reduced mRNA levels of the *AtCHS* gene and was converted into specific siRNAs. In contrast, *NPTII*-dsRNA did not lead to a decrease in *AtCHS* expression, suggesting that *NPTII*-derived sRNAs may have been degraded. Surprisingly, the analysis of the length size distribution of sRNAs for the *AtCHS* and *NPTII* genes revealed some intriguing findings that differed significantly from those of the overall sRNA population [46]. The data demonstrated a clear trend: shorter sRNAs, particularly those around 17 nt in length, exhibited higher read counts compared to their longer counterparts, such as 30 nt sRNAs. The amount of *AtCHS*- or *NPTII*-specific sRNAs decreased gradually when moving from the 17-nt sRNAs to the 30-nt sRNAs, forming a ‘ladder’. Moreover, the distribution of these sRNAs was not uniform across the *AtCHS* and *NPTII* gene sequences. Instead, there were distinct hotspots where read counts peaked, indicating that certain regions of these genes were more actively targeted by the sRNAs. This uneven mapping could imply functional significance, as specific regions may be more critical for the regulation of gene expression.

The existing literature also reveals inconsistencies in the processing efficiency of exogenous RNAs. For instance, Uslu et al. [53] reported a lack of detectable dsRNA processing and subsequent gene silencing of the *GFP* transgene in tobacco plants following foliar application of dsRNA or hpRNA. This suggested insufficient dsRNA uptake by plant cells. However, a subsequent study by the same group [54] demonstrated successful *GFP* silencing in tobacco using high-pressure spraying of three synthetic 22-nt siRNAs that targeted different regions of the *GFP* transgene, indicating the importance of target site selection for optimal RNAi efficacy. Suprun et al. [74] confirmed that target site selection is crucial for optimal RNAi efficacy by treating tomato plants with dsRNA complementary to the *SlTRY* gene promoter, protein-coding region, and intron. This resulted in the highest inhibition of the *SlTRY* gene when targeting the protein-coding region.

A number of studies have employed sRNA-seq techniques to identify virus-specific sRNAs present in plant tissues that have been treated with virus-specific dsRNA, as well as in untreated samples after virus application [43,50,118]. Researchers have also explored the sRNA profiles in plant leaves that received dsRNA targeting specific genes of attacking fungal pathogens [39,111,119]. These investigations demonstrated that the virus-specific dsRNA treatments significantly reduced the levels of virus-derived sRNAs after virus application, thereby enhancing the overall resistance of plants to viral infections or downregulating the expression of target fungal genes. However, our understanding is limited by a lack of information on sRNA profiles resulting from the application of exogenous dsRNAs itself, i.e., independently of viral infection. In a recent high-throughput sequencing (HTS) analysis, Samarskaya et al. [43] investigated non-coding sRNAs as indicators of RNAi triggered by infection with the RNA-containing potato virus Y (PVY) and by the external application of dsRNA corresponding to a segment of the PVY genome. Surprisingly, the externally supplied PVY dsRNA fragment itself (without virus application) resulted in the production of a non-canonical pool of sRNAs, characterized by a ladder-like distribution of sizes ranging from approximately 18 to 30 nucleotides, in contrast to PVY-induced canonical production of discrete 21 and 22 nt sRNA species. This finding suggested the existence of an unexpected sRNA biogenesis pathway that deviates from the conventional mechanisms typically associated with viral infections. Moreover, these non-canonical sRNAs exhibited a limited capacity for systemic movement within the plant and did not promote transitive amplification, which is often a hallmark of effective RNAi responses. Interestingly, this observation aligns with the findings of Nityagovsky et al. [46], who also reported a gradual decline in the distribution of dsRNA-specific sRNAs, spanning from 17-nt to 30-nt lengths, supporting the idea of a ladder-like profile. Understanding how these sRNAs interact with the plant immune system could lead to innovative biotechnological approaches for enhancing crop tolerance or modifying other crop traits.

In summary, these findings highlight the potential for significant advancements in the field of exogenously induced gene silencing in plants. In general, there have been limited research efforts focused on examining how exogenous dsRNA is processed within the plant. Detailed sRNA-seq analyses, coupled with other techniques such as sRNA northern blotting, stem-loop PCR, and other techniques, are needed to unravel the complexities of dsRNA processing in plant cells, which eventually would contribute to the understanding of the exoRNAi mechanism.

## 8. Impact of Exogenous RNAs on Plant Epigenetics

Beyond its well-known role in mRNA degradation, RNAi is now documented to elicit epigenetic alterations [20]. Epigenetic alterations can include changes in DNA methylation patterns, histone modifications, and other processes, including miRNA production [120]. The RNA-directed DNA methylation (RdDM) pathway is a process specific to plants, whereby non-coding RNAs direct cytosine methylation to specific DNA sequences in the plant genome [121]. Epigenetic modifications can have long-lasting effects, persisting long after the initial RNAi event, which raises concerns about potential unintended consequences of exoRNAi. Therefore, it is essential to consider these unintended effects and the specificity of gene silencing when utilizing RNAi technologies.

The broad implications of applying exogenous RNA, particularly with regard to plant epigenetics, remain largely unexplored. Research has shown that suppressing the expression of the *NPTII* and *EGFP* transgenes by applying naked transgene-specific dsRNAs and siRNAs to plant leaves has led to increased DNA cytosine methylation of the transgenes [44,45]. In these studies, the DNA methylation level was analyzed in the transgene coding regions complementary to the applied RNAs. A study by Dalakouras and Ganopoulos [122] has demonstrated that exogenous dsRNAs, particularly those targeting the *Cauliflower Mosaic Virus* (CaMV) 35S promoter of the *GFP* transgene in tobacco plants, induced de novo DNA methylation of the 35S promoter. The observed increase in DNA methylation suggested the involvement of the RdDM pathway in the plant response to exogenous dsRNA. RdDM plays a pivotal role in establishing and maintaining epigenetic states, ensuring that gene expression is finely tuned in response to environmental cues. The RdDM pathway is a complex, evolutionarily conserved mechanism involving RDRs, DCLs, Argonaute proteins, and DNA methyltransferases [20,21]. Exogenous RNA molecules could be involved in this pathway, generating sRNAs that guide the methylation machinery to complementary DNA sequences. However, the precise interplay between exogenous RNA, endogenous sRNA pathways, and the RdDM machinery remains a subject of further research. In addition to DNA methylation changes, exogenous dsRNA has been shown to induce significant alterations in the plant miRNA transcriptome [123]. Foliar application of dsRNA targeting the *AtCHS* gene in *A. thaliana* resulted in extensive changes across the miRNA profile, affecting the expression of 59 distinct miRNAs. In contrast, the application of a non-related bacterial *NPTII*-dsRNA had a minimal impact, influencing the expression of only one miRNA. Expression analysis of some gene targets of the upregulated and downregulated miRNAs documented a negative correlation between the expression of miRNAs and the expression of their predicted targets [123]. The data indicate that exogenous plant gene-specific dsRNAs induce substantial changes in the plant miRNA composition and ultimately affect the expression of a wide range of genes. Thus, the exogenous dsRNAs reshaped the regulatory networks governed by miRNAs, illustrating the intricate interplay between exogenously induced RNAi and plant epigenetics. To the best of our knowledge, no other reports on the epigenetic environment after dsRNA plant treatments have been published.

In summary, exoRNAi influence extends beyond simple gene silencing; it intricately interacts with the plant epigenetic machinery. The potential to manipulate gene expression through RNAi induction, while simultaneously altering epigenetic landscapes, presents both opportunities and challenges. It becomes increasingly clear that a comprehensive understanding of the complexities of RNAi and its broader implications in plant epigenetics is essential for the effective application of RNAi technologies.

## 9. Mechanism of Exogenously Induced RNAi

**Figure 3:**
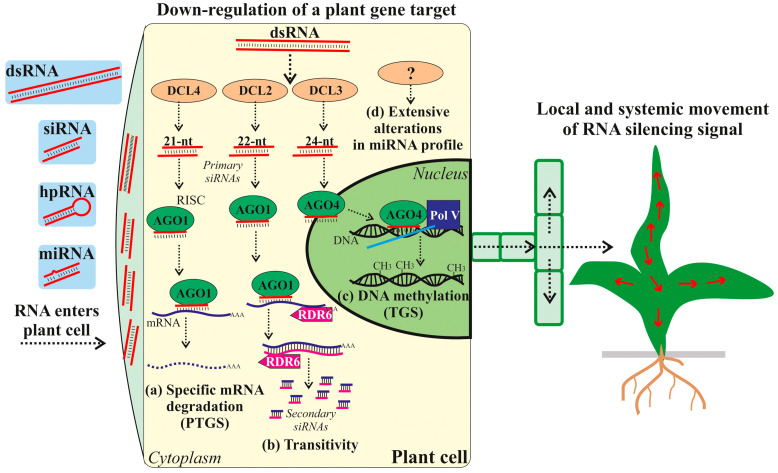
Proposed mechanisms of exogenous RNA interference (exoRNAi) induction and down-regulation of a plant gene target. (**a**) The siRNAs are incorporated into the RISC that guides specific mRNA degradation (PTGS) of homologous mRNAs. (**b**) The components of the siRNA/mRNA complex can be amplified into secondary siRNAs through the activity of RDR6, a process known as transitivity. (**c**) RNA-directed DNA methylation is a mechanism where short RNA molecules guide DNA cytosine methylation to specific sequences, leading to transcriptional gene silencing (TGS). (**d**) Extensive alterations in the plant miRNA transcriptome after exogenous dsRNA application. The dashed arrows depict the different steps of the RNAi induction process and the movement of dsRNA/siRNA at the local level. Red arrows show the systemic movement of the RNA silencing signal in the treated plant. DCL—RNase III enzyme DICER-LIKE; AGO—Argonaute; RISC, RNA-induced silencing complex; RDR—RNA-dependent RNA polymerase; Pol V—DNA-dependent RNA polymerase V.

Currently, our understanding of the molecular mechanisms governing the uptake, processing, and action of exogenous RNAs in plants remains incomplete, largely due to a limited number of comprehensive studies utilizing sRNA-seq and other modern methods. It is reasonable to assume that the absorbed dsRNAs are processed by the plant cellular machinery into functional siRNA molecules within the cytoplasm in order to induce the target gene silencing in the treated plant (Figure 3). The initial cleavage of dsRNA may involve DCL enzymes, a family of RNase III-like enzymes known to be crucial for siRNA generation from dsRNA sources. These siRNAs are key players in the RNAi cascade and presumably interact with the RISC, a protein complex that guides the degradation of target mRNAs, thereby inducing downregulation of the target plant gene (Figure 3a). If this process effectively reduces the levels of target mRNA and, consequently, the corresponding protein, it would lead to observable changes in the plant phenotype.

The efficiency of target mRNA degradation depends on several factors, including the degree of complementarity between the siRNA and the target mRNA, the location of the target site within the mRNA (e.g., 5′UTR, coding sequence, or 3′UTR), and the abundance of the target mRNA itself. For example, a study has shown that siRNAs targeting the 5′ region of the mRNA are often more effective than those targeting the 3′ region, as observed in the silencing of the *GFP* transgene [53,54]. Also, targeting a tomato gene promoter, protein-coding region, and intron revealed the highest inhibition of the gene when targeting the protein-coding region [74].

Beyond the initial silencing, a phenomenon called siRNA amplification or transitivity plays a crucial role in the robustness and spread of the exogenously induced RNAi response (Figure 3b). Uslu et al. [54] also revealed that the exogenous introduction of transgene-specific siRNAs not only silenced *GFP* but also amplified *GFP*-siRNA through the transitivity mechanism [54]. This implies that the initially applied siRNAs triggered the plant endogenous RNAi machinery to generate secondary siRNAs, thereby enhancing the silencing effect. The initial siRNA triggers the plant endogenous RNA-dependent RNA polymerase (RDR) enzymes, such as RDR6, to generate secondary siRNAs from the target mRNA (Figure 3b). These secondary siRNAs then further amplify the silencing effect, leading to more widespread and sustained gene silencing. This amplification contributes to systemic RNAi, where the silencing signal can spread throughout the plant. Research using mutant plants has demonstrated the essential roles of key RNAi components, such as AGO1 and RDR6, in response to exogenous miRNA [38]. Furthermore, exoRNAi influence extends beyond simple gene silencing; it intricately interacts with the plant epigenetic machinery by affecting DNA methylation level (Figure 3c) and miRNA transcriptome (Figure 3d) [44,45,122,123].

Future research is needed to focus on identifying and characterizing additional proteins and pathways involved in the uptake, processing, and spread of exogenous RNAi signals, ultimately leading to more efficient and predictable applications of SIGS/exoRNAi in plant biotechnology.

## 10. Conclusions

Research into exoRNAi induction for plant gene regulation is still in its early stages, but studies have shown that SIGs/exoRNAi can influence plant traits by silencing target genes in the plant genome. These findings suggest that dsRNAs, siRNAs, and miRNAs can be applied exogenously to modify plant growth rate, disease resistance, secondary metabolism, sugar biosynthesis, and stress tolerance. Available studies show that exogenous RNAs can be effective not only in commonly studied plants such as Arabidopsis, tobacco, and rice but also in agriculturally important crops (grapes, tomato, and potato), as well as in rather rare plants (orchids and ginseng). Therefore, the exoRNAi/SIGS method has great potential for application in various fields.

Despite these promising developments, the existing literature on the externally induced silencing of plant gene targets remains limited. This scarcity highlights the need for more comprehensive studies to explore the mechanisms by which exogenous RNAs operate and their long-term effects on plant physiology. There is a need for the investigation of the limitations of SIGs/exoRNAi for plant gene silencing induction, since there are studies reporting on low effectiveness or inconsistency of exogenous RNAs in certain conditions. The efficiency of RNAi may vary greatly depending on several factors, including the plant species, the specific target gene, the method of RNA delivery (e.g., foliar application, root soaking, inoculation, or infiltration), the stability of the exogenous RNA, and potential off-target effects. Future research could focus on optimizing the exogenous delivery of RNAs and on understanding how they interact with the genome of different plant species. Such investigations could contribute to the development of innovative agricultural practices that would enhance crop yields, nutritional value, or create resistant varieties while minimizing reliance on chemical pesticides and fertilizers.

## Abbreviations

CHS	Chalcone synthase
CPP	Cell-penetrating peptides
dsRNA	double-stranded RNA
DCL	Dicer-like proteins
dpt	Days post-treatment
EGFP	Enhanced green fluorescent protein
exoRNAi	Exogenous RNA interference
GFP	Green fluorescent protein
GUS	β-glucuronidase
hpRNA	Hairpin RNA
hpt	Hours post-treatment
LDH	Layered double hydroxide clay nanosheets
lmPEI	Lipid-modified PEI nanoparticles
miRNA	microRNA
MSN	Mesoporous silica nanoparticles
NPTII	Neomycin phosphotransferase II
RISC	RNA-induced silencing complex
RDP	RNA-dependent RNA-polymerase
RdDM	RNA-directed DNA methylation
RNAi	RNA interference or gene silencing
SIGS	Spray-induced gene silencing
siRNA	Small interfering RNA
sRNA	Small RNA
sRNA-seq	Small RNA sequencing
YFP	Yellow fluorescent protein
